# Chinese herbal medicine Shufeng Jiedu capsule for mild to moderate COVID-19: a multicenter, randomized, double-blind, placebo-controlled phase II trial

**DOI:** 10.3389/fphar.2024.1383831

**Published:** 2024-05-27

**Authors:** Chun-li Lu, Liu-qing Yang, Xin-yan Jin, Thomas Friedemann, Yu-fei Li, Xue-han Liu, Xiao-ying Chen, Xiang-yun Zou, Bing-rui Zhang, Fu-xiang Wang, Yuan-long Lin, Yi-min Tang, Meng-li Cao, Ya-lin Jiang, You-fang Gao, Kui Liu, Zhen-gang Tao, Nicola Robinson, Sven Schröder, Jian-ping Liu, Hong-zhou Lu

**Affiliations:** ^1^ Centre for Evidence-Based Chinese Medicine, Beijing University of Chinese Medicine, Beijing, China; ^2^ The Third People’s Hospital of Shenzhen, The Second Affiliated Hospital to Southern University of Science and Technology, Shenzhen, China; ^3^ HanseMerkur Center for Traditional Chinese Medicine at the University Medical Center, Hamburg, Germany; ^4^ The People’s Hospital of Bozhou, Bozhou, China; ^5^ Zhongshan Hospital Affiliated of Fudan University, Shanghai, China; ^6^ Institute of Health and Social Care, London South Bank University, London, United Kingdom

**Keywords:** Shufeng Jiedu capsules, COVID-19, Chinese herbal medicine, symptom relief, randomized control trial

## Abstract

**Background:** The COVID-19 pandemic has had a profound global impact, although the majority of recently infected cases have presented with mild to moderate symptoms. Previous clinical studies have demonstrated that Shufeng Jiedu (SFJD) capsule, a Chinese herbal patent medicine, effectively alleviates symptoms associated with the common cold, H1N1 influenza, and COVID-19. This study aimed to assess the efficacy and safety of SFJD capsules in managing symptoms of mild to moderate COVID-19 infection.

**Methods:** A randomized, double-blind, placebo-controlled trial was conducted from May to December 2022 at two hospitals in China. Mild and moderate COVID-19-infected patients presenting respiratory symptoms within 3 days from onset were randomly assigned to either the SFJD or placebo groups in a 1:1 ratio. Individuals received SFJD capsules or a placebo three times daily for five consecutive days. Participants were followed up for more than 14 days after their RT-PCR nucleoid acid test for SARS-CoV-2 turned negative. The primary outcome measure was time to alleviate COVID-19 symptoms from baseline until the end of follow-up.

**Results:** A total of 478 participants were screened; ultimately, 407 completed the trial after randomization (SFJD, *n* = 203; placebo, *n* = 204). No statistically significant difference in baseline parameters was observed between the two groups. The median time to alleviate all symptoms was 7 days in the SFJD group compared to 8 days in the placebo group (*p* = 0.037). Notably, the SFJD group significantly attenuated fever/chills (*p* = 0.04) and headache (*p* = 0.016) compared to the placebo group. Furthermore, the median time taken to reach normal body temperature within 24 h was reduced by 7 hours in the SFJD group compared to the placebo group (*p* = 0.033). No deaths or instances of serious or critical conditions occurred during this trial period; moreover, no serious adverse events were reported.

**Conclusion:** The trial was conducted in a unique controlled hospital setting, and the 5-day treatment with SFJD capsules resulted in a 1-day reduction in overall symptoms, particularly headache and fever/chills, among COVID-19-infected participants with mild or moderate symptoms. Compared to placebo, SFJD capsules were found to be safe with fewer side effects. SFJD capsules could potentially serve as an effective treatment for alleviating mild to moderate symptoms of COVID-19.

**Clinical Trial Registration**: https://www.isrctn.com/, identifier ISRCTN14236594.

## 1 Introduction

The emergence of the COVID-19 pandemic globally in 2020 led to the predominance of the Omicron variant since November 2021, resulting in milder symptoms due to higher vaccination coverage (World Health Organization, 2021; Iuliano et al., 2022; Wolter et al., 2022; World Health Organization, 2022a; DeWitt et al., 2023). Individuals infected with SARS-CoV-2 Omicron reported various symptoms such as cough, nasal congestion, headache, sore throat, fever or chills, muscle or joint pain, or fatigue (Menni et al., 2022; Sha et al., 2023). Symptomatic management recommended by the World Health Organization (WHO) includes antipyretics, adequate nutrition, and appropriate rehydration (World Health Organization, 2023). However, there is no specific therapeutic recommendation for non-severe COVID-19 patients without risk factors due to insufficient evidence or expensive or potential hazards for drug interactions (World Health Organization, 2022b). Therefore, there is an urgent need for a safe and cost-effective treatment option that can alleviate symptoms associated with a non-severe COVID-19 infection.

Shufeng Jiedu (SFJD) capsule, a Chinese herbal patent medicine, was approved by the China National Medical Products Administration in 2009 (Identifier No. Z20090047) (National Medical Products Administration, 2021). This medicine contains extracts from eight standardized medicinal plants (Supplementary Table S1). In China, SFJD capsules are indicated for treating acute upper respiratory tract infections with symptoms such as fever, sore throat, headache, nasal congestion, runny nose, and cough (Xia et al., 2018). *In vitro* and *in vivo* experiments have demonstrated that SFJD inhibits viral proliferation and attenuates inflammation associated with lung injury (Han et al., 2023). Moreover, clinical trials have employed SFJD capsules for treating respiratory diseases characterized by symptoms such as fever or chills, sore throat, headache, nasal congestion, and cough (Wang et al., 2020; Xia et al., 2020; Chen et al., 2021; Xia et al., 2021; Zhang et al., 2022a). Additionally, pragmatic randomized trials have indicated the potential of SFJD capsules to alleviate COVID-19 symptoms during the Omicron wave (Zhang et al., 2022b), primarily targeting sore throat, cough, fatigue, and fever. Notably recommended in editions four to ten of the “Diagnosis and Treatment Protocol for COVID-19 (Trial)” issued by the China National Health Commission, SFJD capsules have been widely utilized in China for managing COVID-19 patients (National Health Commission, 2020b; National Health Commission, 2020b; National Health Commission, 2020a; National Health Commission, 2020d; National Health Commission, 2020c; National Health Commission, 2020; National Health Commission, 2022b; National Health Commission, 2023). However, to date, there has been a scarcity of randomized clinical placebo-controlled trials with adequate statistical power to assess the efficacy of SFJD capsules specifically in managing mild to moderate symptoms associated with COVID-19.

Therefore, this study aimed to investigate both efficacy and safety aspects through a randomized, double-blinded, placebo-controlled trial assessing symptom relief among patients infected with mild to moderate COVID-19 while adhering to mainland China’s pandemic-related regulations that mandated hospitalization until two consecutive negative SARS-CoV-2 virus polymerase chain reaction (PCR) test results were obtained (Zhang, 2022).

## 2 Materials and methods

### 2.1 Study design

In this multi-center, parallel-group, placebo-controlled, randomized study, a total of 478 participants were initially enrolled between May 2022 and December 2022 from two designated hospitals for COVID-19 admissions and treatment in China: the Third People’s Hospital of Shenzhen, Guangdong, and the People’s Hospital of Bozhou, Anhui.

All enrolled participants tested positive for the SARS-CoV-2 virus through RT-PCR testing and were diagnosed with mild or moderate COVID-19 according to the guidelines set by China (National Health Commission of the People’s Republic of China, 2022). The primary symptoms observed among these participants included fever, dry cough, fatigue, and additional manifestations such as nasal congestion, runny nose, sore throat, loss of smell or taste, conjunctivitis, muscle pain, and diarrhea. Participants exhibiting these symptoms without evidence of pneumonia in imaging examinations like X-rays were classified as having “mild” severity, while those with both aforementioned symptoms and radiographic evidence of pneumonia were labeled as “moderate” severity. To ensure early accuracy and relevancy of SFJD capsules throughout the course of the COVID-19 infection in patients actively displaying signs at registration time within 3 days from symptom onset, only adults aged between 18 and 75 years were considered eligible for participation. Voluntary written informed consent was obtained from all patients before their enrollment.

The trial received approval from the Ethics Committee of the Third People’s Hospital of Shenzhen (Ref: [2022–097]) on 29 April 2022. Prior to enrolling the first participant, it was registered in the ISRCTN registry (Ref. ISRCTN14236594) on May 3.

Following the Chinese Government’s policy on COVID-19 regulation (National Health Commission of the People’s Republic of China, 2022), suspected and confirmed cases were individually isolated in separate inpatient rooms. Close monitoring and daily medication checks were conducted by medical staff. Participants could be discharged if they tested negative for RT-PCR nucleic acid on two consecutive days. Additionally, all participants were required to report their condition and wellbeing through a designated phone app designed specifically for this trial.

### 2.2 Protocol deviations

Protocol deviations were addressed as per the pre-published protocol (Lu et al., 2023). However, due to changes in Chinese COVID-19 regulations starting on 7 December 2022, hospitalization was no longer mandatory for infected patients, resulting in the premature termination of the trial due to insufficient participation at the hospital site. Although the initially planned sample size of 440 patients was not achieved, considering a minimum sample size requirement of 400 with an additional allowance for a potential dropout rate of up to 10%, our current dataset consisting of 407 participants is deemed sufficient for evaluation.

### 2.3 Participants eligibility criteria

1. A positive SARS-CoV-2 virus RT-PCR test was confirmed.
2. The diagnosis of mild and moderate COVID-19 was made according to the guidelines in China, with fever, dry cough, and fatigue being the main manifestations. Some patients also experienced nasal congestion, runny nose, sore throat, loss of smell or taste, conjunctivitis, myalgia, and diarrhea as primary symptoms. Mild severity referred to individuals having relatively mild clinical symptoms without evidence of pneumonia on the imaging examination. Moderate severity included those exhibiting the aforementioned clinical symptoms along with radiographic evidence of pneumonia (National Health Commission of the People’s Republic of China, 2022).
3. Eligible participants were enrolled if they presented with symptomatic COVID-19 at the time of enrollment and had experienced the symptom onset no longer than 3 days prior.
4. The study included adults aged between 18 and 75 years.
5. Patients who have never taken other traditional Chinese medicines for COVID-19 and symptom-releasing and anti-viral drugs within 3 days before enrollment.
6. Participants agreed to take part in this study and accept random allocation.
7. All participants were capable and willing to sign informed consent.
8. Patients were able to provide patient-reported outcome (PRO) data.

### 2.4 Randomization and masking

The random sequence was generated using SAS 9.2 statistical software using blocked random number tables consisting of 440 numbers. Subsequently, the SFJD (*n* = 220) and placebo groups (*n* = 220) were randomly assigned to these 440 numbers. Each eligible participant was then randomized into either the SFJD or placebo group. The process of generating the randomization sequence was separately documented and securely stored in a locked filing cabinet at the Centre for Evidence-Based Chinese Medicine, Beijing University of Chinese Medicine. Trained personnel conducted the randomization procedure while ensuring allocation concealment by appropriately labeling the experimental herbal drugs and placebo. Group assignment remained blinded until the completion of full data analysis.

Participants, clinicians, nurses and care providers, outcome assessors, and data analysts were all kept blinded to the details regarding randomization throughout the study period. To ensure blinding, patients received identical-looking capsules containing either SFJD or placebo formulations that were indistinguishably packaged from each other. Treatment packs were dispatched in two boxes, with allocations based on sequential numbering.

### 2.5 Procedures

Treatment group: participants in the treatment group received SFJD capsules (for constituents, please refer to Supplementary Table S1).

SFJD capsules (Trial capsules Batch No. 3220403) have obtained an over-the-counter drug license in China, adhering to the standardized manufacturing process outlined in the Chinese Pharmacopoeia 2020 (Identifier No. Z20090047 by the National Medical Products Administration, 2021). This formulation comprises eight medicinal herbs: Reynoutria japonica Houtt. [Polygonaceae] (anti-viral), Forsythia suspensa (Thunb.) Vahl [Oleaceae] (anti-viral and cytotoxic), Isatis tinctoria subsp. tinctoria [Brassicaceae] (anti-infective and eliminates toxins), Bupleurum chinense DC. [Apiaceae] (anti-infective and antipyretic), Patrinia scabiosifolia f. scabiosifolia [Caprifoliaceae] (eliminates toxins), Verbena officinalis L. [Verbenaceae] (antipyretic), Phragmites australis subsp. australis [Poaceae] (immunomodulatory), and Glycyrrhiza uralensis Fisch. ex DC. [Fabaceae] (anti-infective and anti-inflammatory).

The SFJD capsules, a licensed Chinese patent medicine, were manufactured in accordance with the Chinese Pharmacopoeia 2020 and underwent standardized chemical analysis. Prior to entering the factory, professional and technical personnel conducted macroscopic and microscopic examinations of the eight medicinal materials, ensuring their conformity with the specifications outlined in the Chinese Pharmacopoeia 2020. A sample of each herb is retained and stored in Anhui Jiren Pharmaceutical Co., Ltd.'s Central Laboratory Sample Retention Room. To ensure adequate levels of three reference compounds (emodin and polydatin from Reynoutria japonica and phillyrin from Forsythia suspensa), high-performance liquid chromatography (HPLC) fingerprinting is performed on samples of all eight herbs at the company’s laboratory. The HPLC-MS method is employed for the identification of mean components.

The manufacturing process is conducted as follows: Reynoutria japonica rhizome and Isatis tinctoria root coarse particles are immersed in 70% ethanol at a volume ratio of 5:1 (ethanol to the ground mixture). Subsequently, the mixture is heated under reflux for 2 hours and then filtered. The resulting sediment is combined with 70% ethanol at a volume ratio of 3:1 (ethanol to sediment), heated under reflux for 1 hour, and filtered again. The filtrates are consolidated, followed by the recovery of ethanol and vacuum concentration into a thick paste with a relative density ranging from 1.35 to 1.40 (at 60°C). Forsythia suspense fruit and Bupleurum root are placed in water for the extraction of volatile oil over 4 hours before being separated through filtration into both filtrate and sediment components. The obtained sediment is further boiled with Verbena, Patrinia, Phragmites rhizome, and Glycyrrhiza uralensis root in water for 2 hours initially, followed by an additional 1-h boiling period. This mixture undergoes filtration and subsequent combination with the filtrate from Forsythia suspense fruit and Bupleurum root prior to vacuum concentration into a thick paste with a relative density ranging from 1.35 to 1.40 (at 60°C). A well-mixed composition comprising dextrin (50 g) and micro-silica gel (50 g) is added to both pastes, which are subsequently subjected to vacuum drying, powderization, and the addition of dextrin until reaching a total weight of 520 g. The volatile oil (diluted with the appropriate amount of absolute ethanol) is sprayed into the mixture. Finally, the volatile oil is diluted with absolute ethanol and then sprayed into the mixture, ensuring thorough sieving and mixing before encapsulation within 1,000 capsules, each containing 520 mg.

Control group: participants in the control group were administered placebo capsules (constituents of corn dextrin (95%) and caramel (5%)). These capsules had a similar appearance, color, and packaging as the SFJD capsule. The placebo capsules were manufactured by the same company, and each capsule contained 520 mg.

All participants took four SFJD or placebo capsules orally three times daily, 30 min after meals, for a duration of 5 days.

### 2.6 Outcomes

The study timeline is presented in Supplementary Table S2. The primary outcome measure was the time for the alleviation of all COVID-19-related symptoms (TTAS) from the start of the trial regimen, during hospital isolation, and on days 7 and 14 during home quarantine. Symptoms were assessed daily using a PRO questionnaire consisting of 16 items that measured symptom severity over the past 24 h on a scale ranging from none to severe (0–3) (Lu et al., 2023). Symptom alleviation was defined as a score of zero for all symptoms on this scale for two consecutive periods of 24 h. TTAS was recorded once patients reported no symptoms for 2 days. Fever clearance time was defined as the time from the first dose of a study drug until the temperature dropped to ≤37.5°C and remained below this temperature for at least 2 days.

The secondary outcomes included the duration of alleviation for individual COVID-19-related symptoms, changes in the proportion of resolved symptoms overall and individually, time taken to negative SARS-CoV-2 virus RT-PCR test results, and time taken for fever alleviation (axillary temperature). Laboratory examination included leukocyte count, lymphocyte count, C reactive protein (CRP) levels, serum procalcitonin (PCT) levels, inflammatory factor (interleukin-6) levels, CD4/CD8 cell ratio, chest CT scans for lung inflammation, as well as assessing participants who progressed from mild or moderate to severe disease by day 6.

The safety assessment involved laboratory examinations, including routine blood tests, coagulation function tests, electrolyte analysis, routine urine tests, an electrocardiogram, and biochemical indicators such as liver enzymes, myocardial enzymes, and kidney function at baseline and on day 6. Any adverse events were recorded daily.

A case report form (CRF) was designed according to the trial protocol and approved by the Ethics Committee. PROs were adapted into a phone application format for easy access. Data collection was facilitated using an electric data capture (EDC) CRF sheet. PRO data were imported through a background service on the phone application, while other outcomes were extracted from the hospital information system. Additionally, a contract research organization (CRO) was engaged for data management purposes and provided a customized remote platform to ensure the implementation of measures for data security and quality control. The unblinding of the data occurred after the completion of the statistical analyses.

### 2.7 Statistical analysis

The sample size calculation assumed an equal distribution of the overall two-sided 5% significance level between the SFJD and placebo groups, as well as a dropout rate of 10%. A sample size of 200 patients in each group was estimated to provide at least 90% power to detect a difference of 1 day in time to alleviate all symptoms, assuming a common standard deviation (SD) of 3 days and a significance level of *p* ≤ 0.05 (Treanor et al., 2000; Bryant et al., 2021; Deng et al., 2021). Taking into account a 10% dropout rate, the target sample size was set at 220 participants per group. The calculations were performed using G*Power 3.1 software (Heinrich Heine University Düsseldorf, Germany).

The data were analyzed using the pre-determined statistical analysis plan (Lu et al., 2023). Statistical analysis involved Kaplan–Meier analysis, hazard ratio estimation, and Wilcoxon Rank tests using SAS software 9.2 (SAS Institute Inc., Cary NC, United States), as well as the generalized estimating equation method for longitudinal data with a binary logistic or Poisson log-linear model. The full analysis dataset (FAS), per-protocol analysis dataset (PPS), and safety analysis dataset (SS) were evaluated based on participants’ adherence. According to the ICH-9 definition for the analysis set, FAS included participants with COVID-19 symptoms who were randomized and took medication at least once, with at least one medical record available. The intervention administered achieved a satisfactory compliance rate of over 80% for medication dosage. Participants were required to complete visits from baseline to day 6, providing valid data for the primary outcome. During the trial period, participants should avoid taking any medications that may affect the evaluation of efficacy, including Chinese herbal medicine with similar efficacy. Baseline characteristics and main outcomes were analyzed in FAS, while missing data were filled using the last observation carried forward approach. PPS was conducted to assess the stability of the results.

## 3 Results

**FIGURE 1:**
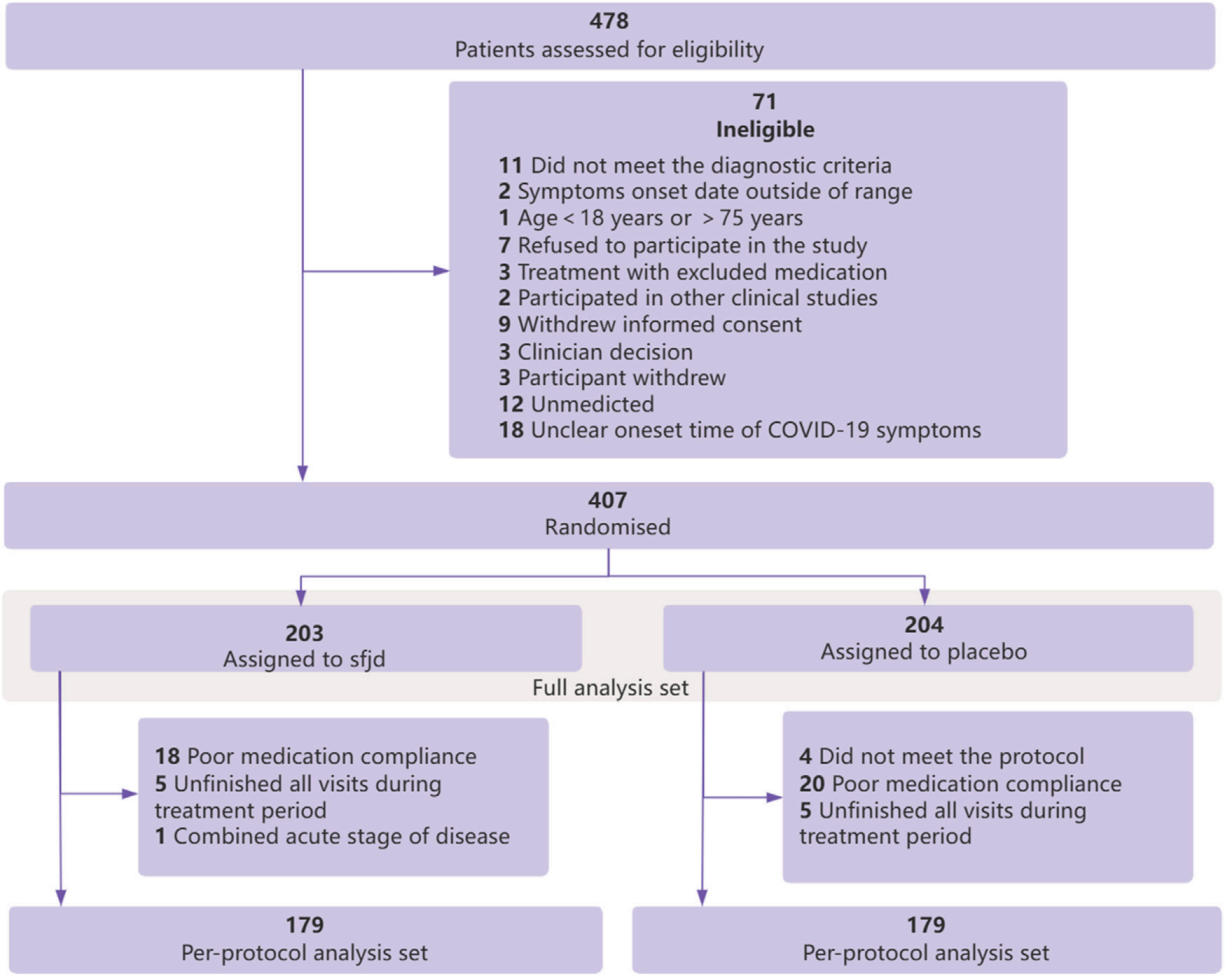
Flowchart of participants.

A total of 478 people were screened for this trial. In addition, 407 participants who met the inclusion criteria and provided signed consent were randomized into the study between 29 May and 8 December 2022. Participants were randomized to the SFJD group (*n* = 203) or placebo group (*n* = 204). Baseline characteristics were balanced (*p* > 0.05), and details are presented as FAS in Table 1 and in the flowchart shown in Figure 1.

**TABLE 1:** Baseline characteristics of participants with COVID-19.

	SFJD group (N = 203)	Placebo group (N = 204)
Age, years (median (IQR))	37 (31–49)	36 (31–47)
Sex		
Female	114 (56.2%)	124 (60.8%)
Male	89 (43.8%)	80 (39.2%)
COVID-19 diagnosis classification		
Mild	201 (99.0%)	200 (98.0%)
Moderate	2 (1.0%)	4 (2.0%)
Vaccination status^a^		
At least one	181 (95.8%)	175 (97.2%)
Never	8 (4.2%)	5 (2.8%)
Education level		
Junior college and below	168 (82.8%)	169 (82.8%)
Undergraduate	29 (14.3%)	31 (15.2%)
Graduate	6 (3.0%)	4 (2.0%)
History of allergies	11 (5.4%)	12 (5.9%)
Smoking history	12 (5.9%)	12 (5.9%)
Taken chest CT scan	137 (67.5%)	131 (64.2%)
Use of antipyretic drugs in 3 days	9 (4.4%)	11 (5.4%)
Clinical symptoms		
Cough	149 (73.4%)	140 (68.6%)
Stuffy nose	64 (31.5%)	73 (35.8%)
Sore throat	79 (38.9%)	77 (37.8%)
Fever or chills	74 (32.2%)	65 (31.9%)
Muscle/joint pain	35 (17.2%)	48 (23.5%)
Headache	45 (22.2%)	63 (30.9%)
Fatigue	45 (22.2%)	49 (24.0%)
Dyspnea	16 (7.9%)	18 (8.8%)
Dry throat	67 (33.0%)	70 (34.3%)
Runny nose	37 (18.2%)	39 (19.1%)
Diarrhea	13 (6.4%)	17 (8.3%)
Lack of appetite	42 (20.7%)	54 (26.5%)
Body aches	24 (11.8%)	30 (14.7%)
Anosmia	11 (5.4%)	16 (7.8%)
Dysgeusia	16 (7.9%)	13 (6.4%)
Nausea	14 (6.9%)	16 (7.8%)

^a^ N is not equal to the total number of the group, as data were unavailable for some of the patients.

### 3.1 Efficacy

**FIGURE 2:**
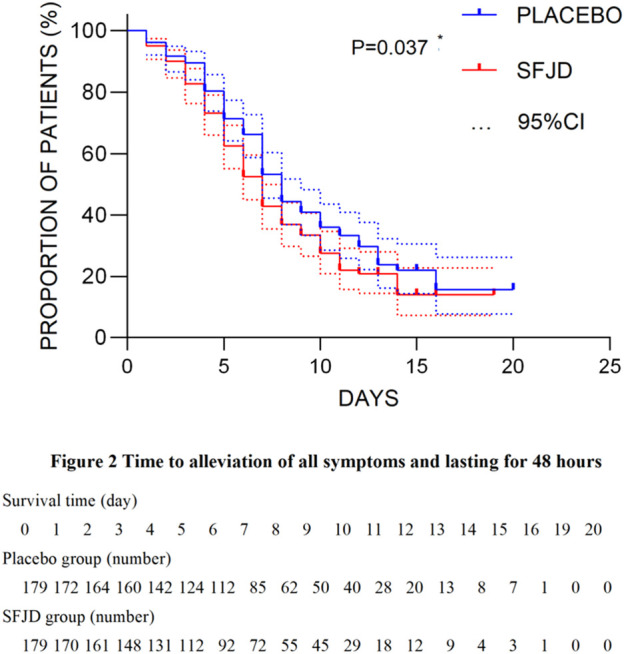
Survival analysis of the time for the alleviation of all symptoms during trial.

The median time to alleviate all symptoms was 7 days in the SFJD group compared with 8 days in the placebo group (*p* = 0.037 by the log-rank test) (Figure 2) and showed a better effect on relieving all symptoms (*p* = 0.017). Details of changes in the proportion of individual symptoms that disappear are given in Supplementary Table S3.

The SFJD group demonstrated a significant improvement in relieving fever or chills (*p* = 0.040, FAS) and headache (*p* < 0.001). Moreover, the median fever clearance time was significantly shortened by 7 hours in the SFJD group (15 h) compared to the placebo group (22 h) (*p* = 0.033). Additionally, there was a notable difference in the level of IL-6 on day 3 between the two groups (*p* = 0.025). Detailed information on all symptoms assessed during the study periods is given in Supplementary Table S3. Importantly, no deaths occurred, symptom severity did not worsen for any patients, and no severe conditions were observed through chest CT scans.

Furthermore, participants did not report any serious adverse events throughout the study duration, and the adverse event rates were 4.4% in each group (Table 2). Notably, there were no reported deaths or severe symptoms among both groups. Statistical analysis revealed no significant difference in the clinical laboratory parameters related to safety or reported adverse events between the two groups, as presented in Supplementary Tables S4, S5.

**TABLE 2:** Adverse events during intervention and follow-up.

Event type	SFJD group (*N* = 203)	Placebo group (*N* = 204)
Stomach ache	0	2
Diarrhea	2	2
Rash	1	1
Hyponatremia	0	1
Hyperhidrosis	1	1
Emesis	1	0
Hemoptysis	0	1
Urinary tract infection	2	0
Hypokalemia	2	1
Total	9 (4.4)	9 (4.4)

Data are presented as no (%).

## 4 Discussion

This study was conducted in a unique setting, which enhances its significance and novelty. First, it adhered to the health policy of the People’s Republic of China during the COVID-19 pandemic, providing a controlled and standardized inpatient hospital environment for observation (Zhang, 2022). Under these controlled circumstances, all participants were subject to professional daily monitoring of symptoms, frequent blood tests, and SARS-CoV-2 RT-PCR tests. This standardized setting ensured consistent and comparable observation conditions for all participants, enabling a robust analysis of the efficacy and safety of the SFJD capsule. The distinctive setting of this study sets it apart from prior trials in other countries where SARS-CoV-2 RT-PCR tests were not conducted as frequently, particularly in asymptomatic cases, leading to challenges in early detection and monitoring of COVID-19 patients. Additionally, as part of mandatory SARS-CoV-2 RT-PCR tests in China (every 1–3 days, depending on local regulations), even asymptomatic cases were identified. Patients involved in this study were detected during the early stages of COVID-19.

The findings of this study demonstrate that the utilization of the SFJD capsule for a duration of 5 days resulted in a reduction of 1 day in the median time required for symptom alleviation. The observed reduction in symptom duration is comparable to the effects observed with other anti-viral medications such as neuraminidase inhibitors (i.e., oseltamivir, peramivir, zanamivir, or laninamivir) or an endonuclease inhibitor (i.e., baloxavir), which has been demonstrated to be effective for patients with uncomplicated influenza (Hayden et al., 2018; Liu et al., 2021). However, medications from these categories and corticosteroids were found to have no impact on the time required for symptom alleviation in COVID-19 patients (López-Medina et al., 2021; Clemency et al., 2022).

Symptomatic management of COVID-19-associated symptoms in the mild Omicron variant of COVID-19 has gained significance due to the rarity of severe symptom development. The major impact of COVID-19 now lies in the duration of milder symptoms and work absenteeism. Therefore, the development of new treatment options that can effectively reduce the duration of symptoms is essential. Furthermore, SFJD capsules exhibited superior efficacy compared to placebo in resolving fever throughout the entire observation phase, clearing fever approximately 7 h earlier than placebo and also alleviating headache more effectively. These are predominant symptoms of the early stage of COVID-19, where SFJD capsules demonstrated their main therapeutic effect (Xia et al., 2021). Alleviating COVID-19 symptoms enables patients to resume their daily activities and return to work or school at a faster pace, thereby enhancing their overall quality of life during recovery. Shortening the duration of symptoms can potentially decrease virus transmission by reducing the likelihood of infected individuals spreading the infection to others.

Compared to the currently available primary registered drugs for COVID-19, nirmatrelvir plus ritonavir (Paxlovid™) stands as the principal registered anti-viral medication. However, its utilization is restricted to patients at a heightened risk of developing severe illness. Previous reviews have highlighted the significant reduction in hospital admission rates and mortality associated with Paxlovid™ (Blair, 2023), yet they have failed to report on the quality of life and symptom resolution (Reis et al., 2023). Therefore, further evidence is required regarding the treatment of mild and moderate COVID-19 infections using interventions such as SFJD capsules that may potentially impact the time taken for symptom alleviation. Paxlovid™ has generally been well-tolerated among adult patients with symptomatic COVID-19 infection. Nonetheless, predominantly mild and moderate adverse events have been reported during or after treatment in 22.6% of cases (Hammond et al., 2022), whereas our study’s SFJD group exhibited mild adverse events in only 4.4% of cases, comparable to both placebo groups and previous studies reporting adverse events over a span of 20 years of pharmacovigilance data on SFJD capsules in China (Zhang et al, 2022a).

Another significant finding from the secondary outcomes is the impact of IL-6. Previous studies have demonstrated that the SFJD capsule effectively reduces inflammatory factors such as IL-6, IL-10, TNF-α, and IFN-γ, leading to a decrease in coronavirus load within lung tissue in a mouse model (Xia et al., 2021). This finding is consistent with the observed reduction of IL-6 levels on day 3 in the SFJD group compared to the placebo group in our study (*p* < 0.05 in PPS; results can be seen in Supplementary Table S4). Additionally, a real-world study has indicated that taking the SFJD capsule earlier leads to faster relief of symptoms (Xia et al., 2021).

In terms of methodology, this trial represents the first prospective, multi-center, randomized, placebo-controlled trial on SFJD capsules specifically designed with rigor in participants diagnosed with mild and moderate COVID-19 caused by the Omicron variant of the SARS-COV-2 virus. The previous studies investigating the symptomatic effect of SFJD capsules for COVID-19 treatment primarily relied on case reports, retrospective studies, and observational studies conducted in a real-world setting. These studies specifically examined participants infected with the Delta variants of the SARS-CoV-2 virus (Wang et al., 2020; Chen et al., 2021; Xia et al., 2021). Therefore, it is important to note that the efficacy evaluation presented in these publications may not directly apply to our current study involving patients infected by the Omicron variant.

All in all, the findings of this study have broader implications for research on herbal medicine and its integration into mainstream healthcare practices. The SFJD capsule serves as a representative example of the growing interest in traditional medicine and the necessity for rigorous scientific investigations regarding its efficacy and safety. Further research on SFJD capsules and other herbal medicines can enhance our understanding of their potential role in COVID-19 treatment, foster collaborations between traditional medicine practitioners and conventional healthcare providers, and promote evidence-based integrative approaches.

One limitation to our study is the change in pandemic control policy that occurred on 7 December 2022 (National Health Commission of the People’s Republic of China, 2022b), which resulted in hospitalization being no longer mandatory. This change led to a sample size that was lower than initially planned. However, it is important to note that we were still able to achieve a sample size of 400 with a statistical power exceeding 90%, indicating the reliability of our results. Furthermore, the unique standardized hospital setting utilized in this study adds to its credibility. Future research should aim to investigate the long-term effects of the SFJD capsule and evaluate its efficacy in treating mild and moderate COVID-19 cases among non-hospitalized patients. Additionally, further randomized controlled trials are necessary to validate our findings and assess how the SFJD capsule compares with other herbal medicines or standard treatments. The need for further research, including randomized controlled trials involving non-hospitalized patients and real-world studies, is essential to confirm its efficacy and safety as well as explore its broader applicability across diverse healthcare settings.

## 5 Conclusion

The SFJD capsule demonstrated a significant impact on symptom relief for individuals with mild and moderate respiratory infections caused by COVID-19, as evidenced by a reduction in disease duration by 1 day in terms of time to alleviate of all symptoms conducted in a unique and controlled hospital setting.

## Data Availability

The original contributions presented in the study are included in the article/Supplementary Material; further inquiries can be directed to the corresponding authors.

## Abbreviations

CRP, C reactive protein; CRF, case report form; CRO, contract research organization; EDC, electric data capture; FAS, full analysis dataset; PCT, serum procalcitonin; PCR, polymerase chain reaction; PPS, per-protocol analysis dataset; PROs, patient reported outcomes; SD, standard deviation; SFJD, Shufeng Jiedu; SS, safety analysis dataset; TTAS, time to alleviation of all symptoms; WHO, World Health Organization.
