# Supplementary material for: Chinese herbal medicine Shufeng Jiedu capsule for mild to moderate COVID-19: a multicenter, randomized, double-blind, placebo-controlled phase II trial
Source: Front Pharmacol. 2024 May 27;15:1383831. doi: 10.3389/fphar.2024.1383831 (PMC11165997; doi:10.3389/fphar.2024.1383831)
Supplement: Supplementary file 1 [file Table1.DOCX]

Supplementary Material

**Chinese herbal medicine Shufeng Jiedu capsules** **for mild to moderate COVID-19: a multicenter, randomized, double-blind, placebo-controlled phase II trial**

[Supplementary Table 1: Consistent of Shufeng Jiedu Capsules 2](#_Toc17591)

[Supplementary Table 2: Schedule of Enrolment, Interventions, and Assessments 4](#_Toc5486)

[Supplementary Table 3: Changes in the proportion of individual symptoms that disappear. 6](#_Toc16307)

[Supplementary Table 4: Inflammatory laboratory parameters in participants with COVID-19 7](#_Toc26396)

[Supplementary Table 5: Laboratory parameters for safety in participants with COVID-19 10](#_Toc19434)

**Supplementary Table 1: Consistent of Shufeng Jiedu Capsules**

| **Botanical species** | **Name in** **Chinese Pharmacopoeia** | **Chinese name** | **Plant part** | **Extraction solvent** | **Proportion** |
| --- | --- | --- | --- | --- | --- |
| *Reynoutria japonica*  Houtt.  [Polygonaceae] | Polygoni cuspidati rhizoma et radix | Hu Zhang | Rhizome | 70% Ethanol | 16.67% |
| *Forsythia suspensa*  (Thunb.) Vahl  [Oleaceae] | Forsythiae fructus | Lian Qiao | Fruit | Water | 13.33% |
| *Isatis tinctoria*  Subsp. *tinctoria* [Brassicaceae] | Isatidis radix | Ban Lan Gen | Root | 70% Ethanol | 13.33% |
| *Bupleurum chinense*  DC. [Apiaceae] | Bupleuri radix | Chai Hu | Root | Water | 13.33% |
| *Patrinia scabiosifolia*  Link [Caprifoliaceae] | Herba patriniaes | Bai Jiang Cao | Herb | Water | 13.33% |
| *Verbena* officinalis L.  [Verbenaceae] | Verbenae herba | Ma Bian Cao | Herb | Water | 13.33% |
| *Phragmites australis*  *Subsp. australis*  [Poaceae] | Phragmitis rhizoma | Lu Gen | Rhizome | Water | 10% |
| *Glycyrrhiza uralensis*  Fisch. ex DC.  [Fabaceae] | Glycyrrhizae radix et rhizoma | Gan Cao | Root | Water | 6.7% |

**Supplementary Table 2: Schedule of Enrolment, Interventions, and Assessments**

|  | **STUDY PERIOD** | | | | | | | | | | | |
| --- | --- | --- | --- | --- | --- | --- | --- | --- | --- | --- | --- | --- |
|  | **Enrolment** | **Allocation** | **Intervention** | | | | | **Continued observation visit** | | | **Follow-up** | |
| **TIMEPOINT** | ***-D1/D0*** | **D0** | ***D1*** | ***D2*** | ***D3*** | ***D4*** | ***D5*** | ***D6*** | ***D7*** | ***D7 or until discharge*** | ***Discharge +7 days*** | ***Discharge +14 days*** |
| **ENROLMENT:** |  |  |  |  |  |  |  |  |  |  |  |  |
| **Eligibility screen** | X |  |  |  |  |  |  |  |  |  |  |  |
| **Informed consent** | X |  |  |  |  |  |  |  |  |  |  |  |
| **Allocation** |  | X |  |  |  |  |  |  |  |  |  |  |
| **Discharge from hospital** |  |  |  |  |  |  |  |  | X | X |  |  |
| **Quarantine at home** |  |  |  |  |  |  |  |  |  |  | X | X |
| **INTERVENTIONS:** |  |  |  |  |  |  |  |  |  |  |  |  |
| ***Shufeng Jiedu capsule*** |  |  |  |  |  |  |  |  |  |  |  |  |
| ***Placebo*** |  |  |  |  |  |  |  |  |  |  |  |  |
| **ASSESSMENTS:** |  |  |  |  |  |  |  |  |  |  |  |  |
| ***Demographic data*** | X | X |  |  |  |  |  |  |  |  |  |  |
| ***COVID-19 symptom scale (PROs)**** |  | X | X | X | X | X | X | X | X | X | X | X |
| ***Body temperature***** |  | X | X | X | X | X | X | X | X | X |  |  |
| ***SARS-COV-2 detection test***  ***(RT-PCR)*** | X |  |  |  | X |  | X | X | X | X | X |  |
| ***Laboratory examination****** | X |  |  |  | X |  |  | X |  |  |  |  |
| ***Chest CT*** | X |  |  |  |  |  |  |  |  | X |  |  |
| ***Safety examination******* | X |  |  |  |  |  |  | X |  |  |  |  |
| ***Adverse events*** |  |  | X | X | X | X | X | X | X | X | X | X |

Notes:

*Clinical symptom score (PROs): All COVID-19 related symptoms including cough, nasal congestion, headache, sore throat, fever or chills, muscle or joint pain, fatigue, shortness of breath, dry throat, runny nose, diarrhea, loss of appetite, body pain, smell, taste, nausea;

**Body temperature: Axillary temperature will be measured 4 times daily (morning, noon, evening and bedtime);

***Laboratory examination: leukocyte, lymphocyte, CRP, serum procalcitonin (PCT), inflammatory factor (interleukin-6), CD4/CD8 cells, blood oxygen saturation;

****Safety examination: blood routine, coagulation function, electrolytes, urine routine, electrocardiogram, biochemical indicators (liver enzymes, myocardial enzymes, kidney function).

**Supplementary Table 3: Changes in the proportion of individual symptoms that disappear.**

|  | **FAS (n=203)** | | | | | | **PPS (n=204)** | | | | | |
| --- | --- | --- | --- | --- | --- | --- | --- | --- | --- | --- | --- | --- |
|  | [Regression coefficient](javascript:;) | [Standard error](javascript:;) | Wald | *P* value | OR (95%CI) | | [Regression coefficient](javascript:;) | [Standard error](javascript:;) | Wald | *P* value | OR (95%CI) | |
| All symptoms | 0.187 | 0.15555 | 1.439 | 0.23 | 1.205 | (0.889, 1.634) | 0.442 | 0.1845 | 5.732 | 0.017 | 1.555 | (1.083, 2.233) |
| Cough | -0.063 | 0.1391 | 0.204 | 0.651 | 0.939 | (0.715,1.233) | -0.009 | 0.146 | 0.004 | 0.949 | 0.991 | (0.744, 1.319) |
| Nose stuffy | 0.251 | 0.1593 | 2.477 | 0.116 | 1.285 | (0.94,1.756) | 0.225 | 0.1664 | 1.832 | 0.176 | 1.253 | (0.904, 1.736) |
| Sore throat | -0.11 | 0.1667 | 0.433 | 0.511 | 0.896 | (0.646,1.242) | -0.119 | 0.1844 | 0.414 | 0.52 | 0.888 | (0.619, 1.275) |
| Fever or chills | 0.424 | 0.2059 | 4.235 | 0.04 | 1.528 | (1.02,2.287) | 0.021 | 0.0169 | 1.595 | 0.207 | 1.022 | (0.988, 1.056) |
| Muscle/joint pain | 0.272 | 0.2163 | 1.583 | 0.208 | 1.313 | (0.859,2.006) | 0.236 | 0.239 | 0.976 | 0.323 | 1.266 | (0.793, 2.023) |
| Headache | 0.477 | 0.1971 | 5.844 | 0.016 | 1.611 | (1.094,2.37) | 2.757 | 0.1475 | 349.427 | <.001 | 15.748 | (11.795, 21.026) |
| Fatigue | 0.13 | 0.2015 | 0.418 | 0.518 | 1.139 | (0.767,1.691) | 0.022 | 0.2067 | 0.011 | 0.915 | 1.022 | (0.682, 1.533) |
| Dyspnoea | 0.09 | 0.3025 | 0.088 | 0.767 | 1.094 | (0.605,1.979) | 0.018 | 0.3202 | 0.003 | 0.955 | 1.018 | (0.544, 1.907) |
| Dry throat | 0.037 | 0.1795 | 0.043 | 0.836 | 1.038 | (0.73,1.476) | 0.021 | 0.1902 | 0.012 | 0.912 | 1.021 | (0.703, 1.482) |
| Runny nose | 0.154 | 0.191 | 0.649 | 0.42 | 1.166 | (0.802,1.696) | 0.114 | 0.2037 | 0.313 | 0.576 | 1.121 | (0.752, 1.671) |
| Diarrhea | -0.22 | 0.1958 | 1.267 | 0.26 | 0.802 | (0.547,1.177) | -0.26 | 0.2145 | 1.47 | 0.225 | 0.771 | (0.506, 1.174) |
| Poor appetite | -0.665 | 0.1704 | 15.205 | <.001 | 0.515 | (0.368, 0.719) | 0.031 | 0.1898 | 0.027 | 0.87 | 1.032 | (0.711,1.496) |
| Body ache | 0.256 | 0.2572 | 0.994 | 0.319 | 1.292 | (0.781,2.139) | 0.237 | 0.2758 | 0.74 | 0.39 | 1.268 | (0.738, 2.177) |
| Dysosmia | -0.221 | 0.2708 | 0.667 | 0.414 | 0.802 | (0.471,1.363) | -0.609 | 0.2667 | 5.219 | 0.022 | 0.544 | (0.322, 0.917) |
| Dysgeusia | -0.889 | 0.2378 | 13.99 | <.001 | 0.411 | (0.258,0.655) | -0.005 | 0.0223 | 0.044 | 0.834 | 0.995 | (0.953, 1.04) |
| Nausea | 0.172 | 0.2677 | 0.412 | 0.521 | 1.187 | (0.703,2.006) | 0.006 | 0.0034 | 3.232 | 0.072 | 1.006 | (0.999, 1.013) |

**Supplementary Table 4: Inflammatory laboratory parameters in participants with COVID-19**

|  | | FAS（n=407） | | | PPS（n=358） | | |
| --- | --- | --- | --- | --- | --- | --- | --- |
|  | | SFJD group | Placebo group | *P* value | SFJD group | Placebo group | *P* value |
| WBC (109/L) | Day 0 | 5.31 (4.07 to 6.63) | 4.990 (3.895 to 6.350) | 0.3838 | 5.30(4.06 to 6.64) | 4.81(3.87 to 6.40) | 0.1944 |
|  | Day 3 | 4.70 (3.88 to 5.93) | 4.67 (3.665 to 5.69) | 0.2839 | 4.445(3.880 to 5.150) | 4.195(3.485 to 5.335) | 0.5153 |
|  | Day 6 | 5.43 (4.41 to 6.57) | 5.415 (4.480 to 6.415) | 0.9051 | 5.765(4.990 to 6.890) | 5.53(4.70 to 6.72) | 0.2388 |
| NE# (109/L) | Day 0 | 3.36 (2.23 to 4.65) | 3.095 (2.155 to 4.285) | 0.4027 | 3.34(2.22 to 4.67) | 2.98(2.07 to 4.27) | 0.2116 |
|  | Day 3 | 2.4 (1.69 to 3.43) | 2.27 (1.630 to 3.245) | 0.1859 | 2.07(1.51 to 2.58) | 1.920(1.410 to 2.565) | 0.4764 |
|  | Day 6 | 2.85 (2.09 to 3.77) | 2.835 (2.125 to 3.595) | 0.8841 | 2.965(2.320 to 3.830) | 2.845(2.190 to 3.590) | 0.3523 |
| LY# (109/L) | Day 0 | 1.16 (0.87 to 1.57) | 1.115 (0.81 to 1.515) | 0.2921 | 1.90 (1.45 to 2.22) | 1.11(0.80 to 1.55) | 0.7718 |
|  | Day 3 | 1.60694 (1.110 to 2) | 1.60694 (1.115 to 2.02) | 0.9607 | 1.90 (1.45 to 2.22) | 1.845(1.525 to 2.195) | 0.7718 |
|  | Day 6 | 2.01 (0.74) | 2.06 (0.78) | 0.5141 | 2.24 (1.86 to 2.61) | 2.17(1.82 to 2.73) | 0.8057 |
| NE% (%) | Day 0 | 64.8 (52.4 to 72.5) | 63.55 (51.9 to 72.35) | 0.7923 | 65.1(52.2 to 72.8) | 62.8(51.6 to 72.5) | 0.6343 |
|  | Day 3 | 53.67(14.37) | 51.84(14.54) | 0.2016 | 45.82±10.18 | 44.63±10.46 | 0.4105 |
|  | Day 6 | 52.40(11.96) | 51.91(11.55) | 0.6731 | 50.87±9.79 | 50.52±9.43 | 0.7726 |
| LY% (%) | Day 0 | 23.3 (16.2 to 33.5) | 23.40 (16.35 to 34.55) | 0.7874 | 22.7(16.2 to 34.1) | 23.7(16.4 to 35.3) | 0.9626 |
|  | Day 3 | 35.001(23.6 to 44.40) | 36.35(26.10 to 47.05) | 0.2248 | 42.02±9.74) | 43.40±10.28 | 0.3246 |
|  | Day 6 | 37.01(11.75) | 37.59(12.09) | 0.6236 | 37.5(33.0 to 45.6) | 40.0(33.8 to 46.7) | 0.4012 |
| CRP (mg/L) | Day 0 | 6.55 (2.84 to 12.25) | 7.305 (3.845 to 11.88) | 0.4596 | 6.22(2.84 to 12.25) | 6.47(3.55 to 11.66) | 0.8562 |
|  | Day 3 | 4.67(1.44 to 8.91) | 3.965(1.36,8.51) | 0.6524 | 2.645(1.200 to 7.790) | 2.59(0.83 to 6.47) | 0.2715 |
|  | Day 6 | 1.85(0.63 to 5.38) | 1.55(0.5 to 4.81) | 0.2221 | 1.21(0.45 to 2.11) | 0.970(0.340 to 2.345) | 0.5195 |
| PCT (ng/mL) | Day 0 | 0.029 (0.02 to 0.059) | 0.032 (0.02 to 0.0575) | 0.5698 | 0.027(0.020 to 0.062) | 0.028(0.020 to 0.055) | 0.8246 |
|  | Day 3 | 0.02(0.02 to 0.045) | 0.0205(0.02 to 0.04) | 0.8750 | 0.020(0.020 to 0.043) | 0.020(0.020 to 0.031) | 0.1107 |
|  | Day 6 | 0.02(0.02 to 0.03) | 0.02(0.02 to 0.0325) | 0.8785 | 0.020(0.020 to 0.020) | 0.020(0.020 to 0.020) | 0.7052 |
| IL-6 (pg/mL) | Day 0 | 7.384 (5.309 to 9.712) | 7.9325 (5.4365 to 10.9) | 0.4446 | 7.291(5.278 to 10.220) | 7.629(5.240 to 11.120) | 0.8167 |
|  | Day 3 | 6.510(4.636 to 8.223) | 6.1820(3.7005 to 9.152) | 0.7783 | 6.234(3.834 to 7.936) | 4.410(2.152 to 7.083) | 0.0253 |
|  | Day 6 | 4.212(1.586 to 7.315) | 3.511(1.5 to 7.8285) | 0.9431 | 2.692(1.500 to 5.543) | 1.908(1.500 to 4.715) | 0.2664 |
| CD4/CD8 | Day 0 | 1.47 (1.11 to 1.89) | 1.415 (1.145 to 1.835) | 0.8738 | 1.46(1.09 to 1.90) | 1.390(1.145 to 1.905) | 0.9110 |
|  | Day 3 | 1.52(1.16 to 1.9) | 1.485(1.190 to 1.92) | 0.9610 | 1.57(1.26 to 1.95) | 1.53(1.25 to 1.93) | 0.9668 |
|  | Day 6 | 1.57(1.21 to 1.97) | 1.555(1.21 to 1.935) | 0.7539 | 1.57(1.27 to 2.05) | 1.61(1.26 to 1.95) | 0.8378 |

Abbreviations: WBC: White Blood Cell Count; NE#: neutrophil count; LY#:lymphocyte count; NE%:neutrophilic granulocyte percentage; LY%:Lymphocyte percentage; CRP:C-reactive protein; PCT:procalcitonin; IL-6:Interleukin-6; CD4/CD8: cluster of differentiation 4/cluster of differentiation 8.

**Supplementary Table 5: Laboratory parameters for safety in participants with COVID-19**

|  |  | **SFJD group (n= 203)** | **Placebo group (n=204)** | ***P* value** |
| --- | --- | --- | --- | --- |
| AST (U/L) | Day 0 | 30 (24.50 to 37.60) | 29.15 (24.80 to 34.35) | 0.4507 |
|  | Day 6 | 28.3 (23.90 to 34.60) | 28.25 (23.60 to 33.30) | 0.4292 |
| ALT (U/L) | Day 0 | 22 (14 to 38) | 19.5 (14 to 30) | 0.1898 |
|  | Day 6 | 21 (14 to 36) | 20 (15 to 30) | 0.2873 |
| ALP (U/L) | Day 0 | 69.1 (57.3 to 80.9) | 70.6 (61.55 to 79.00) | 0.8170 |
|  | Day 6 | 66.6 (56.4 to 79.1) | 66.85 (56.05 to 76.20) | 0.736 |
| TBIL (μmol/L) | Day 0 | 10.6 (7.6 to 15.1) | 9.4 (6.45 to 12.90) | 0.0622 |
|  | Day 6 | 10.1 (7.1 to 13.9) | 9.60 (6.65 to 13.10) | 0.2602 |
| BUN (mmol/L) | Day 0 | 3.75 (3.09 to 4.28) | 3.725 (2.995 to 4.325) | 0.7767 |
|  | Day 6 | 3.73 (3.08 to 4.34) | 3.785 (3.045 to 4.430) | 0.8043 |
| UA (μmol/L) | Day 0 | 340.966 (276.3 to 409.2) | 329.3 (267.60 to 380.65) | 0.0452 |
|  | Day 6 | 324.173 (269.8 to 384.1) | 312.7 (256.9 to 370.45) | 0.018 |
| SCr (μmol/L) | Day 0 | 67.2 (57.5 to 77.6) | 69.65 (57.65 to 83.35) | 0.3521 |
|  | Day 6 | 62.9 (55.3 to 74.3) | 65.65 (53.95 to 77.115) | 0.7671 |
| LDH (U/L) | Day 0 | 170.01 (146 to 191) | 164 (147.5 to 179) | 0.0560 |
|  | Day 6 | 161.574 (141 to 182) | 156 (137 to 173.5) | 0.0178 |
| CK (U/L) | Day 0 | 72 (49 to 103) | 66.5 (50 to 93.5) | 0.5637 |
|  | Day 6 | 51 (39 to 74) | 45.5 (35 to 65) | 0.0339 |
| CKMB (ng/mL) | Day 0 | 0.22 (0.22 to 0.36) | 0.22 (0.22 to 0.28) | 0.5847 |
|  | Day 6 | 0.22 (0.22 to 0.22) | 0.22 (0.22 to 0.22) | 0.1541 |

Abbreviations: AST: aspartate transaminase; ALT: alanine transaminase; ALP: A Lkaline Phosphatase; TBIL: total bilirubin; DBIL: direct bilirubin; BUN: Blood Urea Nitrogen; UA: uric acid; SCr: serum creatinine; LDH: lactate dehydrogenase; CK: creatine kinase; CKMB: creatine kinase-MB; K+: kalium; Na+: natrium; Cl-: chlorine; Ca2+ calcium.
